# Metals and mineral phases of dusts collected in different urban parks of Krakow and their impact on the health of city residents

**DOI:** 10.1007/s10653-017-9934-5

**Published:** 2017-03-14

**Authors:** Alicja Kicińska, Piotr Bożęcki

**Affiliations:** AGH University of Science and Technology, Kraków, Poland

**Keywords:** Mineral phases, Dust, Urban parks, Health

## Abstract

The authors present the results of chemical and mineralogical analyses of urban dusts collected in the spring seasons of 2015 and 2016 in three different parks of the Cracow agglomeration. The parks are located in the city centre, in the Nowa Huta industrial district and in a new housing development situated around 9 km west of the city centre. Mineralogical instrumental analyses included the SEM, FTIR and XRD methods and revealed that the dusts of Cracow are highly amorphous and contain significant amounts of hydrocarbons, whereas quartz, feldspars, kaolinite and gypsum are their crystalline phases. Chemical analyses were carried out using the ICP-MS method on aqua regia extracts of the starting samples. The contents of selected toxic elements are: As 5–123; Cd 1–14; Pb 56–258; Zn 486–1891 mg/kg and Fe 0.74–4.02 wt%. The health risk of these elements imposed on the residents of Cracow frequently visiting the three urban parks was assessed on the basis of the health quotient index HQ. At its values exceeding 1, adverse health effects are probable in humans. The HQ values calculated for As and Tl contained in the Cracow Park dusts in the case of adults are 3.42E−01 and 3.00E−01, respectively. They are significantly higher (one order of magnitude) in the case of children 3.19E+00 and 2.27E+00, respectively.

## Introduction

A civilization expansion of urban agglomerations gives not only considerable advantages but also a whole range of hazards (Skotak et al. 2012). One of the latter is contamination of the environment with toxic compounds of heavy metals (among others of Cd, Pb and Zn), metalloids (for instance As), and some organic and inorganic substances, which occur in air, water and soil. Their presence in the environment seriously threatens the health of living organisms. With regard to a human population, the health aspects include mainly reproductive problems, mental disorders, allergies, asthma and tumour incidences (Goudarzi et al. 2016; Khaniabadi et al. 2016). The WHO reported that environment-related diseases were responsible in 2010 for 15–20% of the total mortalities in Europe (Europe Environment 2010). The major reasons were identified as the suspended dusts PM_10_ and PM_2.5_, radonium, tobacco smoke and noise. Introducing technological improvements aimed at reducing emissions of dusts (mostly of the industrial origin) have brought about the expected effects in Europe, with regard particularly to the air pollution with SO_2_, CO and NO_x_. However, in large agglomerations there are still growing numbers of vehicles used by public and private transport, and also they are a significant source of emissions of heavy metals (such as Cr, Ni, Zn and Pb) resulting from fuel combustion and normal exploitation wear (Kicińska 2016a, b; Mazzei et al. 2008; Samek et al. 2016). It is a problem faced in most large towns located on almost all continents (WHO 2016). In the northern latitudes, the problem is not only a nuisance but becomes dangerous particularly in winter months, in which the permitted values of the PM_10_ and PM_2.5_ dusts are often exceeded even more than several times (Boldo et al. 2006; Moreno et al. 2011; Yu et al. 2013; Zhang et al. 2015).

Therefore, investigations of urban dusts within the area of Krakow, one of larger and the oldest European agglomerations, were carried out in the years 2015–2016 focusing on:

1. determination of the total contents of selected metals (Cd, Fe, Hg, Ni, Pb, Tl, Zn) and a metalloid (As) related to exploitation of vehicles and combustion of solid fuels. They belong to the group of the elements of high toxicity, particularly to younger population;
2. identification of the dominating mineral phases that are carriers of selected metals in the urban dusts collected in three parks of Krakow;
3. establishing whether the metal-bearing forms occurring in the urban dusts are of the natural or anthropogenic origin.

The results of the chemical determinations were the basis of evaluating the health risk of the people spending much time in urban parks where they can be endangered due to a prolonged exposition to heavy metallic elements. This analysis was conducted calculating the so-called hazard quotient (HQ).

## Research area

The Krakow agglomeration covers 327 square km, of which 4.3% are forested. Its number of permanent population in 2014 was 762,000 at the positive birth rate 0.6‰ (GUS 2015). During 10–11 months, the city is additionally inhabited by around 300,000 students. Each year Krakow is also visited by tourists and other newcomers: in 2015 slightly above 10 million people (foreigners exceeding 50%) paid short visits to Krakow (www.krakow/pl). The city plays a cultural and educational role but is also an important industrial centre. In the last 10 years, the production manufactured and sold by various enterprises of Krakow has jumped from 12,928 to 28,044 million PLN (GUS 2015), proving a dynamic increase in this sector. An efficient existence of any larger agglomeration requires the presence of a passable road network, whose length in the Krakow area in 2014 was 294 km per 100 km^2^. If compared with the respective data for 2005, the total length of city roads and streets increased by a mere 7% in 2014 (from 898 to 960 km, respectively), whereas the number of cars licensed in the Małopolska Voivodeship (Krakow is its capital town) by more than 50%. These are mainly passenger cars, which in Krakow make 76% of all the vehicles owned by town residents. Adding a long-distance movement of vehicles and the lack of ring roads, there is nothing strange that major thoroughfares are overloaded not only during rush hours.

In Krakow, there are 24 enterprises particularly affecting the air quality: 13 of them are equipped with the installations reducing dust contaminants and only two with the installations cleaning gaseous contaminants. In 2015, nine of them did not have internal stations measuring dust emissions and seven the emissions of gases, while emissions (i.e. overall deposition of contaminants from any own and outer sources) was measured on the premises of three of them (GUS 2015).

The total of dust emissions within the Krakow area in 2012 was 1900 Mg (4400 Mg in 2005), and out of it the dusts generated by combustion of car fuels made 0.900 Mg. The gaseous contaminants, excluding CO_2_, reached 28,500 Mg, broken down into 32% SO_2_, 42% CO and 22% NO_x_, accompanied by H_2_S, N_2_O, NH_3_, HCl, HF and hydrocarbons. Although almost 99% of the dusts were caught in dust-reducing installations, only 1.1% gaseous products were stopped from going into the air. The quality of the air of Krakow is a serious issue and a subject of many papers (e.g. Rzeszutek et al. 2014; Kicińska and Klimek 2017; Samek et al. 2016) and reports of specialized state units (e.g. www.krakow.pios.gov.pl). Part of respective reports is devoted to the impact of such factors as the diameter of aerosol particles, their concentration and chemical composition on the health of the Krakovians. Some contaminants inhaled with the air may be dissolved in body fluids (in bronchial mucus), and after entering the blood circulation system they reach other organs. The process is particularly dangerous in the prenatal period of a child development. Research of Jędrychowski and its team in the years 2000–2004 shown that the newborns whose mothers lived in Krakow throughout the whole pregnancy were (on the average) lighter by 128 g and shorter by 0.9 cm and had their head perimeter shorter by 0.3 cm in comparison with those born in non-polluted regions. The so-called whistling breathing is more often observed in the children with a lower birth body mass, which is a significant herald of possible asthmatic problems in the future. In a further development of such youngsters, the following findings were noted (Jedrychowski et al. 2008): lower values of their lung expiratory volume by around 100 ml, recurrent infections of the respiratory system (five times higher frequency of bronchi inflammation), and lower parameters of their psycho-motoric development (an average of 3.8 points on the scale of the intelligence quotient index IQ).

Despite introducing many preventive and reducing measures to control the quantity of pollutants in the Krakow air, the results of its monitoring still arise much anxiety of the city residents (Table 1). The statistics for 2010–2015 quote for some districts as many as 200 days per year in which the permissible air levels of NO_2_, suspended PM_10_ and PM_2.5_ particulates, and the content of the B(a)P [benzo(α)pyrene] in the suspended PM_10_ particulates were exceeded. Seasonal variations of the PM_2.5_ concentration of Cl, K, Br, Pb, Cu and Zn were reported by Samek et al. (2016). They also identified seven indicators (pollutants) that allow attributing a single pollution source to each one of them. The sources distinguished (op. cit.) are: steel industry, traffic (diesel and gasoline exhaust gases), road dusts, construction dusts, soil dusts, combustion of coal and/or biomass, non-ferrous metallurgical industry.

**Table 1:** Air quality parameters in Cracow in May 2015 and 2016 (data of the State Air Monitoring, Krakow 2015–2016)

Location of the monitoring stations^a^	Year	Parameter	SO_2_ (µg/m^3^)	NO_2_ (µg/m^3^)	NO_x_ (µg/m^3^)	NO (µg/m^3^)	CO (µg/m^3^)	C_6_H_6_ (µg/m^3^)	PM_10_ (µg/m^3^)	PM_2.5_ (µg/m^3^)
Al. Krasickiego Park no. I	2015	Average^d^	–	70	208	90	697	–	45	27
		Min.–max.	–	51–88	106–297	36–149	376–972	–	27–60	17–40
	2016	Average	–	64	189	82	715	1.1	46	28
		Min.–max.	–	38–84	91–274	34–137	448–971	0.6–1.8	23–94 (10)^c^	14–47
Nowa Huta Park no. II	2015	Average	6.2	27	44	11	455	1.5	31	19
		Min.–max.	2.3–13.3	8–42	9–79	1–29	261–726	0.6–2.6	14–53 (1)^c^	9–33
	2016	Average	5.4	23	37	9	449	1.3	25	16
		Min.–max.	1.5–10.0	11–40	11–88	0–34	276–842	0.4–3.0	13–46	8–36
Skawina Park no. III	2015	Average	7.4	19	26	5	–	–	25	–
		Min.–max.	1.4–22.7	10–28	12–43	1–13	–	–	13–37	–
	2016	Average	4.2	18	24	4	–	–	25	–
		Min.–max.	1.5–14.9	9–25	11–42	1–13	–	–	15–37	–
Upper limits during^b^										
1 h			350	200		nd	nd	nd	50	nd
8 h			nd	nd		nd	10,000	nd	nd	nd
24 h			125	nd		nd	nd	nd	nd	nd
1 year			20	40		30	nd	5	40	20

“–” not measured

“nd” no data

^a^Monitoring stations located in the nearest distance from the urban parks considered

^b^According to Regulation of the Minister of the Environment on the level of some substances in the air. Dz. U. poz.1031

^c^The number of days in which the upper limits were exceeded

^d^Monthly average

## Materials and methods

**Fig. 1:**
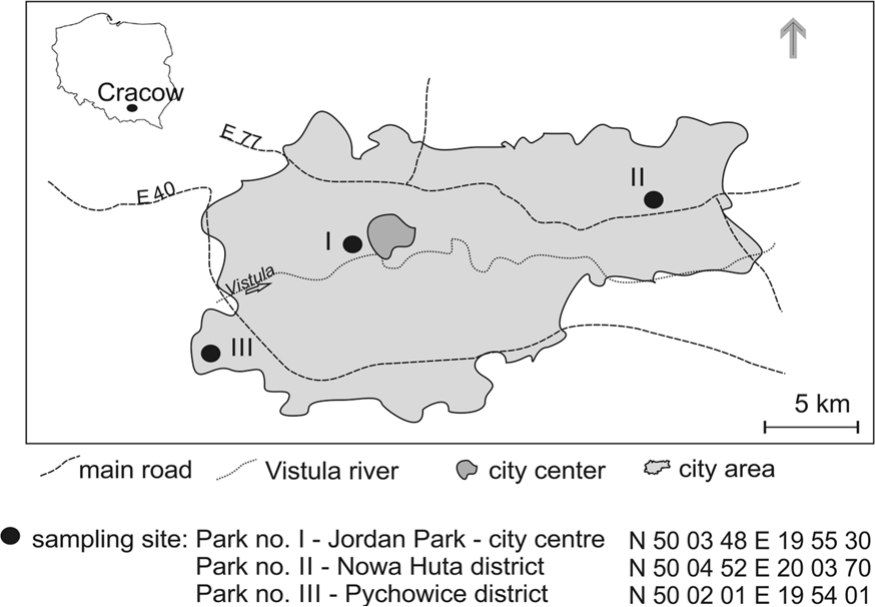
Location of sampling sites within the limits of the Cracow city

The material investigated represents urban atmospheric dusts collected in the spring months (May) of 2015 and 2016 in three city parks numbered I–III (Fig. 1). The selection of sampling sites was based on the results published by Samek et al. (2016) and pertaining to the contents of major and trace element in the PM2.5 during different seasons of the 2014/2015 period in Krakow. In the winter time, the amount of airborne dusts increases due to combustion of coal and biomass fuels, which results in increasing emissions of Cl, K, Cr, Mn, Cu, Zn, Br, Rb and Pb. However, because of weather conditions children do not spent much time in urban parks. In contrary, the emissions are lower in summer months, but children frequently visit and play in the parks and it may be assumed that the health risks during winter and summer months are comparable. Accordingly, also comparable should be the spring and autumn.

In every park, nine dust samples with a weight around 100 mg each were collected from outer surfaces of recreational and sport facilities by rinsing them with distilled water (“wet method”) and by brushing (“dry method”). The two techniques were applied because of the research approach. The samples designated to chemical determinations were collected with the “wet method” by rinsing only those parts of the playing facilities that come into the contact with children hands: it has been assumed that only this part of dusts should be considered in HQ calculations. The solutions were next evaporated to dry mass on a water bath and the solid samples used for chemical determinations. The samples to be studied with the XRD, SEM and FTIR methods were collected as a free-falling dust deposited from the ambient air on single-stage impactors lined with a paper filter (“dry method”).

Park no. I: The Jordan Park represents the city centre and is located around 2 km from the historic Main Market. The area around has the highest traffic load of moving vehicles, and the park itself is one of the oldest and most popular resting places of the Krakow residents.

Park no. II: The Nowa Huta Park represents the industrial part of Krakow and is located around 1 km from the ArcelorMittal Poland plant. The plant is a metallurgical facility (formerly the Tadeusz Sendzimir Steelworks) producing annually around 1.3 mln Mg of steel. Its annual emissions are: 2.7 mln Mg of CO_2_; 2407 Mg of NO_x_; 4236 Mg of SO_x_ and above 450 Mg of suspended dust (Sustainability Report 2014).

Park no. III: Located in a modern housing development of Pychowice, a district distant at around 9 km to SW of the city centre.

The methods of X-ray diffractometry (XRD), Fourier transformed infrared spectroscopy (FTIR) and scanning electron microscopy (SEM) were used to determine the phase composition of dust particles, and SEM also to observe the surface morphology. Applying a one-step extraction of metals with concentrated acids (HNO_3_ + HCl + HClO_4_) at a ratio of the solid phase (dust) to the liquid phase (mixture of acids) of 1:10, sample eluates were obtained. Their chemical composition was determined using the method of induced coupled plasma mass emission spectroscopy (ICP-MS) at the certified hydrogeochemical laboratory (certificate of the Polish Accreditation Commission no. AB1050) of the AGH University of Science and Technology in Krakow. The precision of the determining Fe, Ca, Al, K, Mg, Si, Mn, Na, Zn, Ba, Pb, As, Ti, Cd, Ni, Tl, Cr and Co was 10%, while the accuracies ranged between 95 and 105%. The limits of detection (LOD) and quantification (LOQ) parameters were calculated from the following equations:

1 $${\text{LOD}} = X_{\text{b}} + 3\,{\text{SD}}_{\text{b}} ,$$

2 $${\text{LOQ}} = X_{\text{b}} + 10\,{\text{SD}}_{\text{b}} ,$$

where *X*_b_, mean concentration of the blank (zero concentration) sample; SD_b_, the standard deviation of the blank; and their values are presented in Table 2.

**Table 2:** Chemical composition of dust samples from Krakow

Element	LOD (mg/dm^3^)	LOQ (mg/dm^3^)	Park no. I	Park no. II	Park no. III	All dust samples	
			Min.–max. *n* = 9	Min.–max. *n* = 9	Min.–max. *n* = 9	Min.–max. *n* = 27	*x* ± SD
Main					(wt%)		
Fe	0.0094	0.0176	2.73–2.92	0.74–4.02	1.11–1.53	0.74–4.02	2.17 ± 1.1
Ca	0.0213	0.0314	1.13–1.78	0.41–1.69	0.49–0.55	0.41–1.78	1.01 ± 0.6
Al	0.0071	0.0091	0.89–0.98	0.25–0.41	0.32–0.34	0.25–0.98	0.53 ± 0.3
K	0.2506	0.2848	0.34–0.56	0.06–0.17	0.11–0.14	0.05–0.56	0.23 ± 0.1
Mg	0.0029	0.0049	0.44–0.66	0.11–0.66	0.18–0.22	0.11–0.66	0.38 ± 0.2
Si	0.0083	0.0674	0.79–3.44	0.04–0.15	0.07–0.15	0.04–3.44	0.78 ± 1.2
Trace					(mg/kg)		
Na	0.0620	0.1099	1079–2826	96–837	504–692	96–2826	1006 ± 869
Zn	0.0290	0.1159	907–1335	561–1891	486–558	486–1891	956 ± 509
Mn	0.0014	0.0034	524–758	216–687	284–329	216–758	467 ± 205
Ba	0.0064	0.0213	191–1403	44–843	69–151	44–1403	450 ± 505
Ti	0.0004	0.0011	359–479	18–256	101–129	18–479	224 ± 159
Pb	0.0047	0.0463	201–204	56–208	213–258	56–258	190 ± 63
As	0.1046	0.2655	76–123	5–31	105–107	5–123	75 ± 43
Cr	0.0016	0.0064	68–87	13–85	65–67	13–87	64 ± 24
Ni	0.0065	0.0196	29–56	8–30	15–226	8–226	61 ± 75
Co	0.0038	0.0097	20–52	3–140	2–39	2–140	43 ± 47
Tl	0.0120	0.0421	bdl–18	bdl–1.4	33–53	bdl–53	17 ± 20
Cd	0.0013	0.0029	6–14	1–5	5–6	1–14	6 ± 4
Hg	0.0054	0.0340	bdl–4.7	bdl–0.6	bdl–3.7	bdl–4.7	1.5 ± 2

*LOD* limit of detection, *LOQ* limit of quantitation, *x* arithmetic average, *SD* standard deviation for the whole population (*n* = *27*), *n* number of samples, *bdl* below the detection limit

Statistical calculations and data presentation were conducted with the Statistica version 10 and Excel applications. The group analyses were based on variables (concentrations of elements) characterising the objects considered (i.e. parks) and allowed distinguishing the groups (clusters), inside which the parameters selected are more similar within the given group than to the parameters within other groups. This type of the analysis allows detecting whether the groups reveal any regularity (correlation) and reduces the databases to the averages calculated for specific groups. The Ward method is an analysis of a variance problem, based on minimizing totals of square deviations within the groups (clusters). The method accumulates into clusters the cases with minimum diversifications.

The phase composition of crystalline components was carried out applying the powder Debye–Scherrer method using a Rigaku MiniFlex 600 XRD diffractometer. The measurement parameters were as follows: CuK_α_ radiation, reflection graphitic monochromator, lamp voltage 40 kV, lamp current 20 mA, recording range 2°–72°2*Θ*; step 0.05°2*Θ*, impulse count rate 1 sek/step. The interplanar distances obtained from the X-rays patterns were used for identifying crystalline phases based on the data of the ICDD (International Centre for Diffraction Data) catalogue and the XRAYAN software.

The infrared absorption spectra were recorded within the range from 4000 to 400 cm^−1^ with a resolution of 4 cm^−1^ using a FTIR model FTS 165 spectrometer (made by BIO-RAD). The samples had a form of tablets pressed at 10 MPa from a powdered mixture of 0.5 mg of the sample and 200 mg of spectrally pure potassium bromide (KBr).

The quantity of hydrocarbons was determined with a FTIR spectrometer model FTS 165. The samples measured were organic extracts obtained by treating starting samples with tetrachloroethylene at a solid-to-liquid ratio of 1:20. The determinations were carried out according to the DIN 38409, H. 18 standard. The concentrations of aliphatic and aromatic hydrocarbons are based on the intensities of respective absorption bands of the CH_2_, CH_3_ and *C*_arom._ groups.

The morphology of dust components was determined using a FEI Quanta model 200 FEG scanning electron microscope. The observations were extended by chemical analyses of microareas applying an EDS detector (SEM–EDS method) conducted in the high vacuum mode. The resolution power of the microscope was increased by covering the samples with carbon prior to analysing. The accelerating voltage was 20 kV.

The health risk index HQ from a prolonged exposition to heavy metals was calculated using the equation of Leung et al. (2008) as HQ = ADD/RfD, where ADD—average daily dose, and RfD—reference dose (in mg/kg per day). The ADD value was calculated from the following equation (Wcisło 2009; US EPA 1986):

3 $${\text{ADD}} = \frac{{C \cdot {\text{lngR}} \cdot {\text{EF}} \cdot {\text{ED}}}}{{{\text{BW}} \cdot {\text{AT}}}} \cdot {\text{CF}}1,$$

where *C* mean heavy metal concentration in the dust (mg/kg); IngR, conservative estimates of dust ingestion rates, accepted for children as 200 mg per day and for adults as 100 mg per day; EF, exposure frequency, accepted as 350 days/year; ED, exposure duration, accepted as 6 years for children and 70 years for adults; BW, body weight, accepted as 15 kg for children and 70 kg for adults; AT, averaging time, accepted as 6365 days for children 6 years old and 70,365 days for adults 70 years old.CF1, unit conversion factor of 10^-6^.

The values HQ ≤ 1 suggest a low probability of health risk, the values HQ > 1 mean a probability of negative health effects. The values HQ > 10 are considered high, resulting in a chronic health risk caused by exposition of toxicants.

## Phase composition and morphology of the urban dusts of Krakow

### X-ray diffraction analyses

**Fig. 2:**
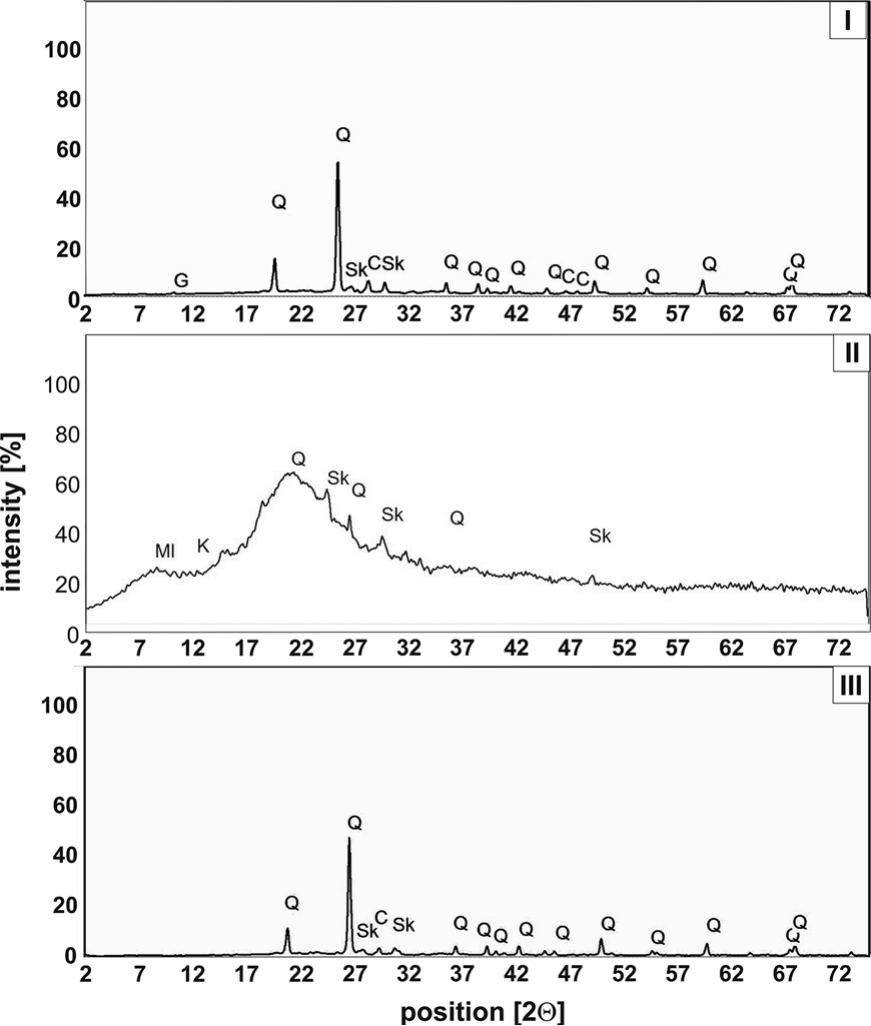
XRD patterns of urban atmospheric dust samples collected in: I—Jordan Park, II—Nowa Huta Park, III—Pychowice Park (Q—quartz, Sk—feldspar, C—calcite, Ml—muscovite, G—gypsum)

X-ray diffraction of the dust fallouts shows a substantial differentiation of their crystalline components (Fig. 2). The samples from the Jordan Park (Fig. 2I) and Pychowice (Fig. 2III) contain highly crystalline mineral phases: quartz SiO_2_, which is the major mineral of both sites, sodium–calcium feldspars (plagioclases) Na[AlSi_3_O_8_]–Ca[Al_2_Si_2_O_8_] and potassium feldspars K[AlSi_3_O_8_], accompanied by minor amounts of calcite CaCO_3_. Additionally, gypsum CaSO_4_·2H_2_O is present in the sample from the Jordan Park (park no. I).

The X-ray patterns of the airborne dusts from the district Nowa Huta (park no. II) have significantly elevated diffraction backgrounds (Fig. 2II), which indicates the presence of a highly amorphous material. Amorphisation of the particulate matter usually proves its anthropogenic origin. Of the crystalline phases, quartz was identified without any doubts, and probable trace minerals include feldspars and clay minerals of the kaolinite group (Al_4_[Si_4_O_10_](OH)_8_).

Silicate and aluminosilicate components (quartz and feldspars) are mostly primary minerals of the geogenic origin. The prevalence of quartz may be explained by blowing away psammitic–aleuritic grain fractions (2–0.1 mm) of sand from nearby sandboxes that are a typical playing facility of city parks.

The remaining, minor mineral phases were identified as carbonates (mainly calcite) and sulphates (fine-grained gypsum). The latter is a typical secondary component of airborne dusts formed due to the so-called salt weathering, i.e. the reaction of sulphuric acid (derived from atmospheric SO_2_) with carbonate minerals.

### Infrared spectroscopic analyses (FTIR)

**Fig. 3:**
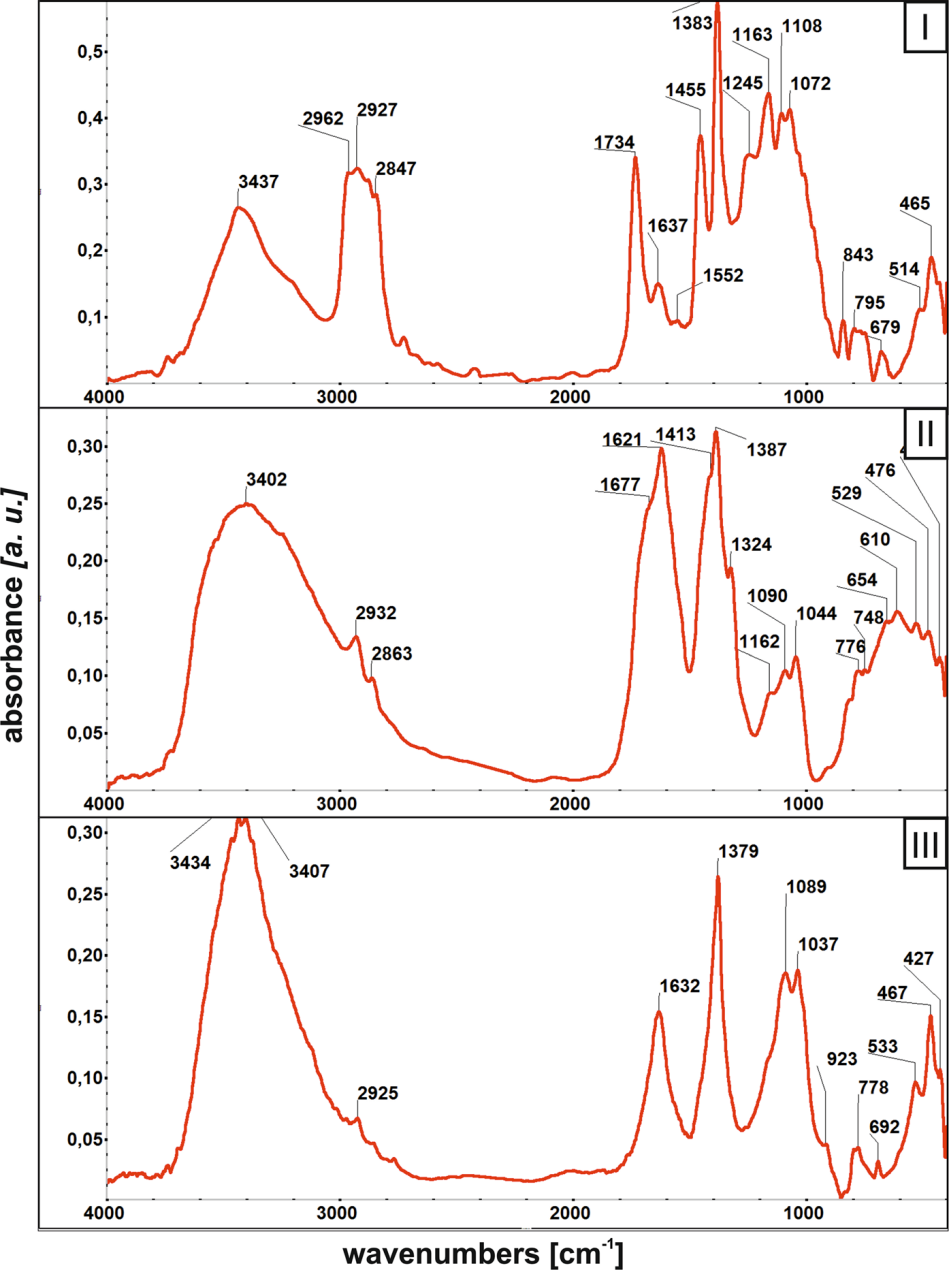
FTIR spectra of the urban atmospheric dust samples, I—Jordan Park; II*—*Nowa Huta Park; III*—*Pychowice Park

The spectra of the samples collected in the Jordan Park (Fig. 3, no. I) show a considerable contamination of dusts with hydrocarbons, whose diagnostic, intensive absorption bands occur between 2960 and 2850 cm^−1^. The presence of hydrocarbons most probably results from a considerable movement of vehicles in this city area. Among mineral phases, the dusts of the Jordan Park contain gypsum (absorption bands 1160 and 1100 cm^−1^), quartz/opal (intensive absorption bands around 1070, 770 and 460 cm^−1^) and muscovite/illite (intensive absorption bands around 3440, 1630, 1080, 520 and 465 cm^−1^).

Calcite (absorption bands around 3440, 1420 and 700 cm^−1^) and gypsum (absorption bands 1620, 1160, 1100 650 and 600 cm^−1^) have been identified in the spectra of the dust samples of the park in Nowa Huta (Fig. 3, no. II). These mineral phases are accompanied by minor amounts of quartz and layered aluminosilicates of the muscovite/illite type. The dusts also contain traces of hydrocarbons, marked by distinct bands in the range 2930–2860 cm^−1^.

The phase composition of dust samples collected in the park in Pychowice (Fig. 3, no. III) is comparable to that of the Jordan Park. Mineral phases include quartz, calcite and layered aluminosilicates of the muscovite/illite type, all of them most probably of the geogenic origin. The dusts of Pychowice contain traces of hydrocarbons, occurring in the lowest quantities of the three parks considered. Low quantities of these oil-derived contaminants are a proof of low pollution of the Pychowice area by the motor traffic.

### Hydrocarbons in urban dusts (FTIR analyses)

Extraction of airborne dusts with tetrachloroethylene aimed at the quantification and identification of hydrocarbons released into the atmosphere by combustion of motor fuels. The highest amounts (6927 mg/kg) were found in the dusts of the Jordan Park. It is mainly the mineral oil (6924 mg/kg), accompanied by traces of benzine (3.3 mg/kg). In the dust collected in Nowa Huta, the total of hydrocarbons was 4.5 times lower (1564.15 mg/kg): the mineral oil contributes 1563.722 mg/kg, while benzine occurs in traces (0.435 mg/kg). The dust content of hydrocarbons was the lowest in Pychowice: the mineral oil prevails (1047.8 mg/kg), while benzine occurs in traces (0.2 mg/kg).

The contents of hydrocarbons in the dust fallouts compared with the limits of hydrocarbons in soils (the Regulation of the Minister of the Environment of 2002) indicate a significant contamination of the parks with oil-derived substances. The limit of benzines in the soils of the A-type areas, i.e. those under the legal protection, is 1 mg/kg, and of mineral oils 30 mg/kg. The remaining soils of groups B and C have the upper limits of benzines 1–750 mg/kg and of mineral oils 50–3000 mg/kg, depending on the depth of soil sampling and water permeability of the soils.

### Scanning electron microscopy (SEM–EDS)

The SEM images and point EDS chemical determinations confirm that the dusts contain both the geogenic material, mainly quartz grains, and the substances of the secondary, anthropogenic origin, i.e. hydrocarbons and gypsum.

**Fig. 4:**
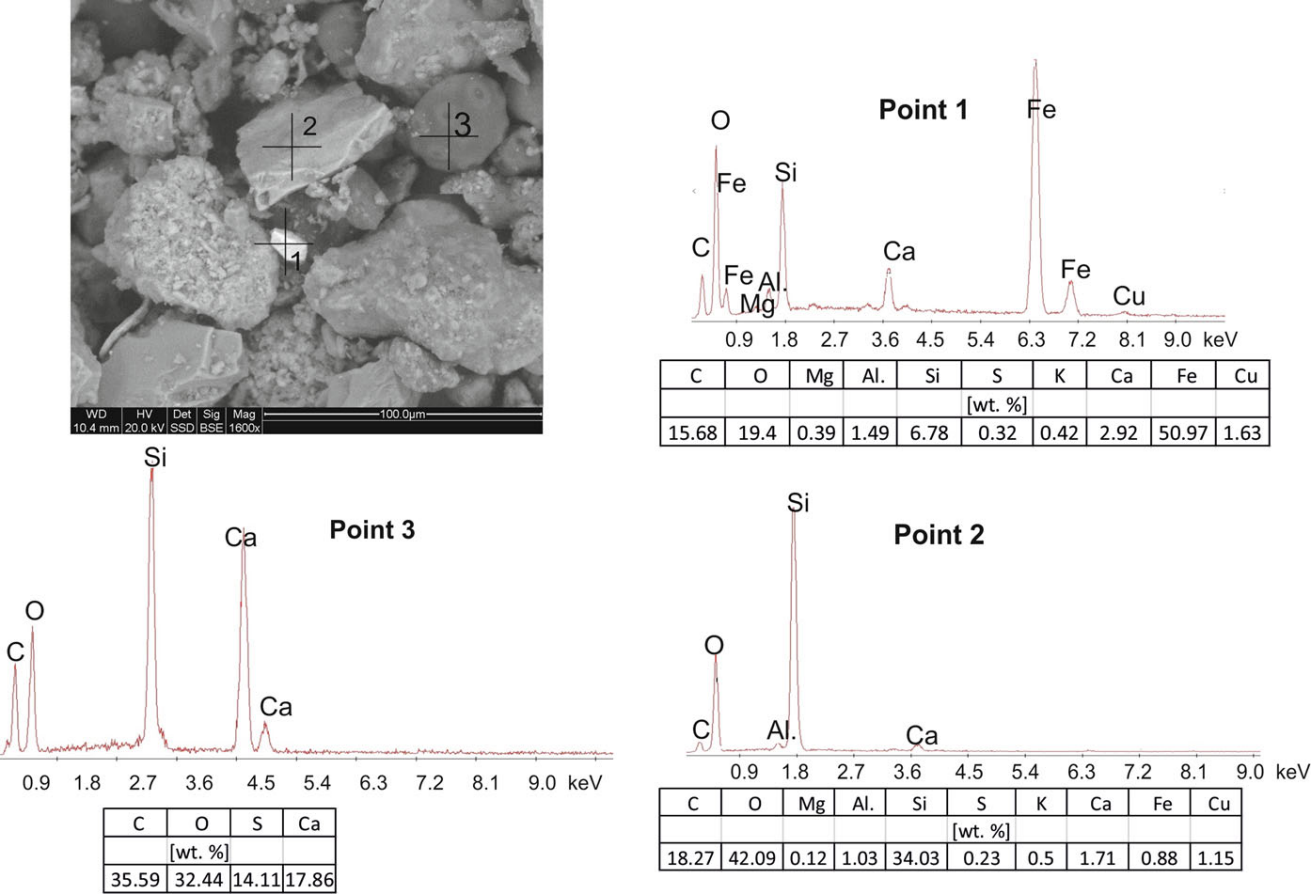
SEM image with EDS spectra of the dust particles collected in the Jordan Park (2016)

The dusts collected in the Jordan Park (Fig. 4) reveal the presence of large grains (20–100 µm), angular with irregular shapes, and of spherical and oval forms with diameters of around 10–20 µm. They are interspersed in a very fine (<1 µm) carbonate-siliceous mass. The irregular grains represent mainly quartz, a few of them possibly also feldspars (point 2, Fig. 4). Some of them are coated with very fine-grained (0.5–1 µm) prismatic or acicular, secondary gypsum crystals. The spherical forms (point 3, Fig. 4) are fragments of partly burned coal and are signs of deposition of dusts attributable to the low emission. Their contribution to the total number of the particulate matter is rather large. The spheres are also often covered with fine-crystalline gypsum crystals. A grain smaller (5–10 µm) than the other particles and differing from them due to its white colour in the SEM image (point 1, Fig. 4) represents an aggregate composed of oxides and hydro-oxides of iron.

**Fig. 5:**
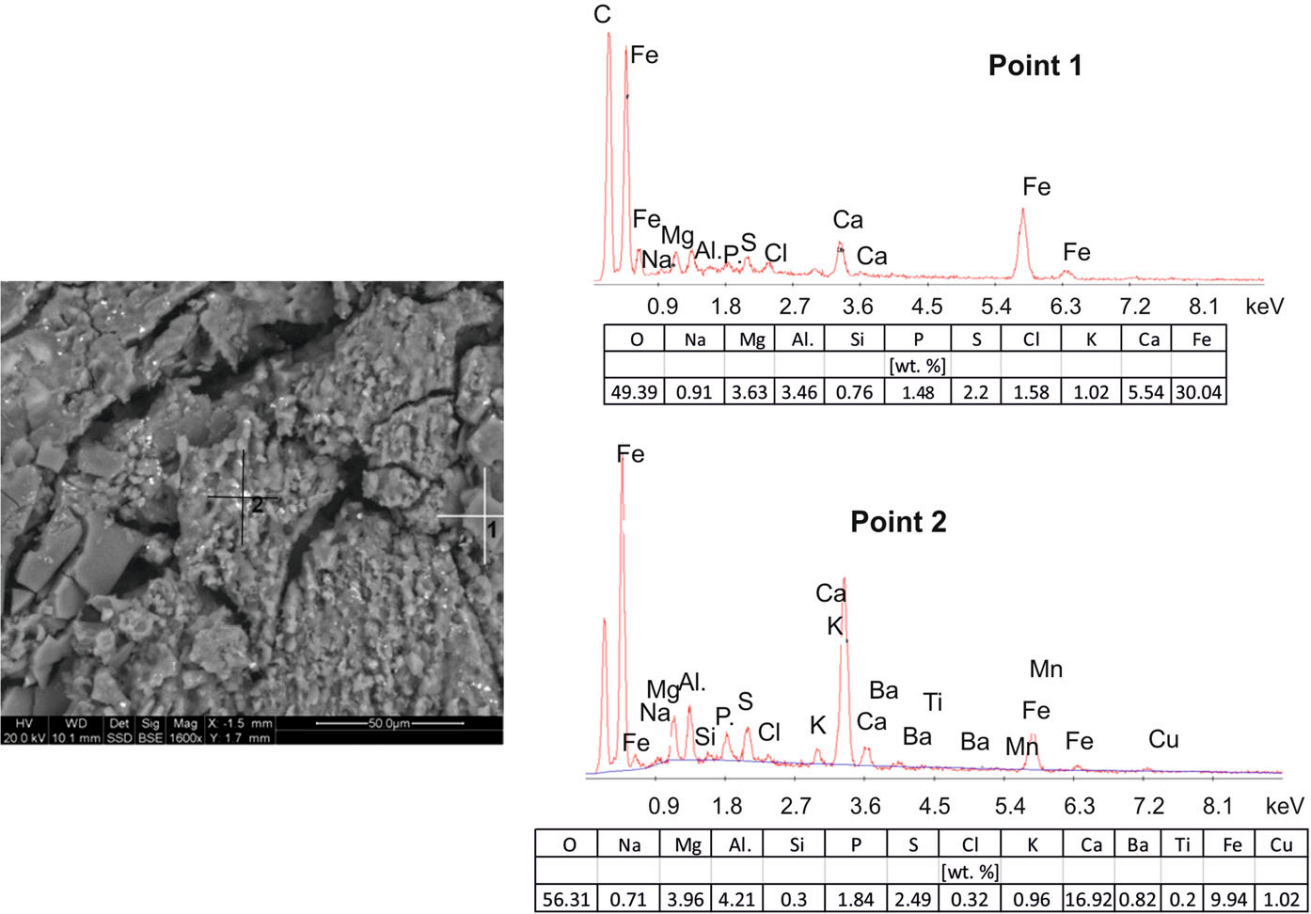
SEM image with EDS spectra of the dust particles collected in the park in Nowa Huta (2015)

The dust particles of the Nowa Huta Park show a considerable fragmentation of quartz and feldspar grains (Fig. 5) that are dominating components. These autogenic silicate and aluminosilicate minerals are covered with fine needles of gypsum. Larger quartz grains have precipitation covers composed of cryptocrystalline mixtures mainly of Fe and Mn and of minor Mg oxide/hydroxides (point 1, Fig. 5). In many places were observed small metallic microspherules with the diameters below 5 µm consisting mainly of Fe alloys with admixtures of Pb and Ba (point 2, Fig. 5). The metallic character indicates their origin in operations of the metallurgical plant located not far from the sampling site.

**Fig. 6:**
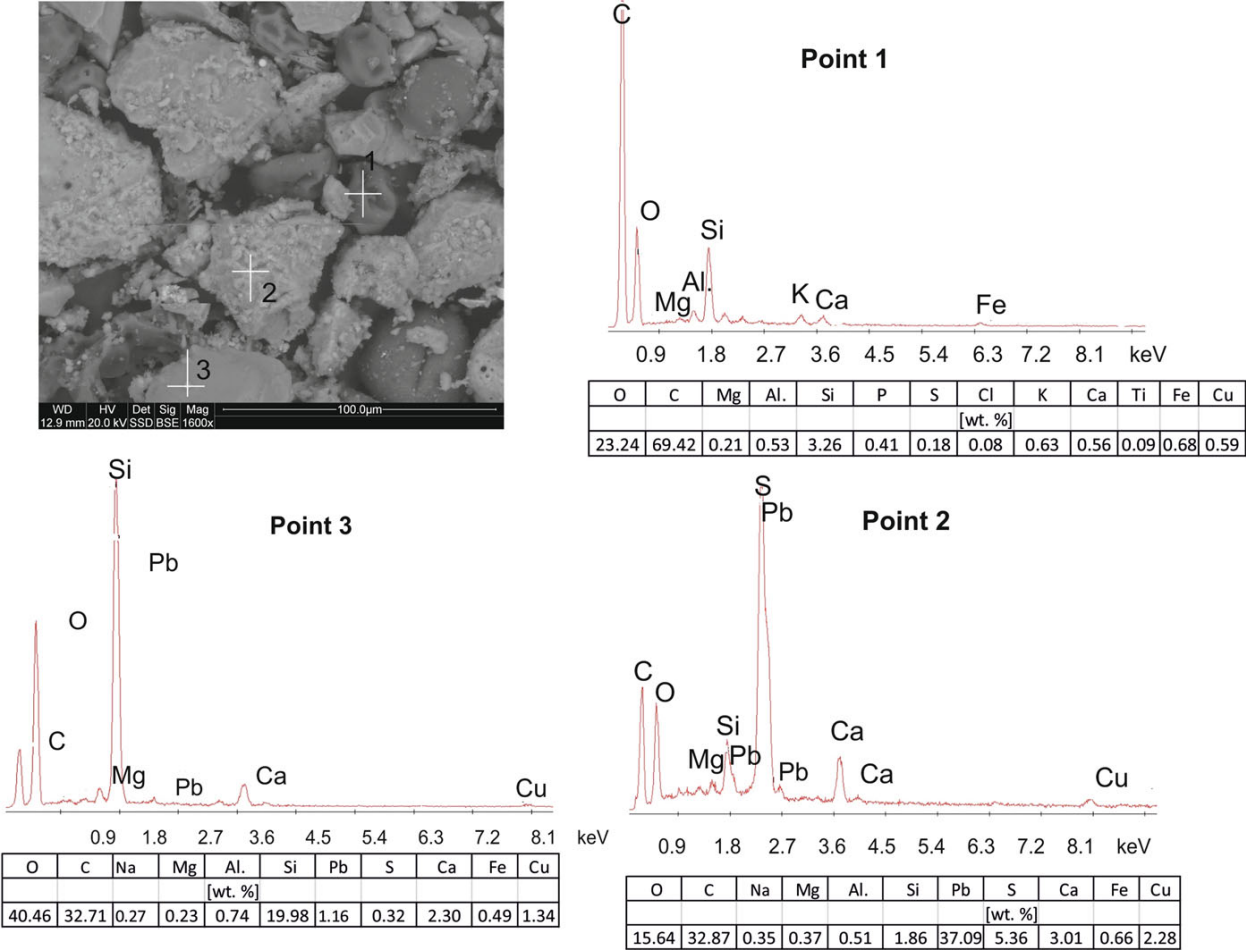
SEM image with EDS spectra of the dust particles collected in the park in Pychowice (2016)

The dust particles collected in the Pychowice Park also have diversified sizes (Fig. 6). Larger grains (30–60 µm) represent quartz and minor feldspars (points 2 and 3, Fig. 6), often covered with fine needles of gypsum. Spherical grains of smaller sizes (diameters 10–20 µm), composed entirely of carbon, are products of unburned coal (point 1, Fig. 6). They are usually covered with tiny grains (<1 µm) of other components, some of them being cryptocrystalline mixtures of oxides and/or oxide/hydroxides of various metals, mainly of Fe and Mn.

## Chemical composition of the urban dusts of Krakow

Applying extraction of dust samples with a mixture of strong acids, the ICP-MS analysis provides data on an overall composition of the dust. A particular attention in current determinations was paid to selected heavy metals/metalloids that even if occurring in trace amounts are toxic to living organisms.

The dominant elements in the acid extracts of dusts of the Jordan Park, i.e. close to the city centre (Table 2), are of the rock-forming type: Ca, Al, K, Mg and Si. Their quantities in the dusts collected in the park no II of the industrial Nowa Huta district are distinctly lower. The proximity of the Arcelor steel works is confirmed by the highest contents of Fe (0.74–4.02 wt%). Such a distribution of the elements was previously reported by Kicińska-Świderska (1999) and Samek et al. (2016). The lowest quantities of the rock-forming elements occur in the extracts from the Pychowice park area (site no. III), being three times lower than those of the Jordan Park, (site no. I).

The mean contents of Fe, Ca, Si, Al, Mg and K: 2.17, 1.01, 0.78, 0.53, 0.38 and 0.23 wt%, respectively, were calculated for all samples of each of the parks. The most differ the Si values: their variability coefficient *V* is 1.53. In the case of the remaining rock-forming elements, their *V* values are between 0.43 and 0.59. The method applied, which suits best the extraction of metallic elements, is insufficient to break down completely silicates and aluminosilicates; therefore, the quantities of Si and Al should be treated as largely underestimated.

Selected metallic and metalloid trace elements (Ba, Cd, Co, Cr, Hg, Mn, Na, Ni, Pb, Ti, Tl, Zn and As) represent a particular group in environmental considerations. Most of them are toxic, and even their low concentrations cause illness symptoms, while at higher concentrations they may be lethal. The so-called death group is composed of As, Tl, Cd and Pb, all of them being carcinogens. The airborne park dusts contain As in the range between 5 and 123 mg/kg (the mean of all the samples is 75 mg/kg). The highest As contents (up to 123 mg/kg) occur in the dusts of the Jordan Park (park no I) located in the city centre, medium ones in a modern housing district of Pychowice (up to 107 mg/kg), and the lowest ones (5–31 mg/kg) in the industrial district Nowa Huta. Comparable dust contents of Pb with their mean 190 mg/kg were found in all three parks; however, the highest lead pollution range—213–258 mg/kg—occurs in the Nowa Huta Park. The same trend is shown by the pollution with Ni and Tl. The presence of these metals is associated with heavy traffic of vehicles: the combustion of car fuels and a normal exploitation wear being major reasons (Mazzei et al. 2008). Of other elements, the dust Cd contents are the highest in the Jordan Park (6–14 mg/kg), the lowest in the park in Nowa Huta (1–5 mg/kg) and the park in Pychowice (5–6 mg/kg). Except lead, the contents of two other metals in airborne dusts are the highest in the park of the Nowa Huta industrial district: 561–1891 mg/kg Zn and 3–140 mg/kg Co.

The general load of metals contained in the airborne dusts is the heaviest in the town centre, which refers to Mn, Na, Ba, Ti, Cd, Cr and Hg. The pollution is a result of an almost continuous movement of vehicles along major Krakow thoroughfares and the low emissions, i.e. the emissions caused by burning hard coal and coal products in households and small boiler houses (Samek et al. 2016). The industrial district Nowa Huta has its air loaded with the dusts carrying Zn and Co, the elements attributed to steel-making operations. The dust contents of Pb, Tl and Ni in the living district Pychowice, elevated in respect to other districts, must be linked to an intensive traffic.

**Fig. 7:**
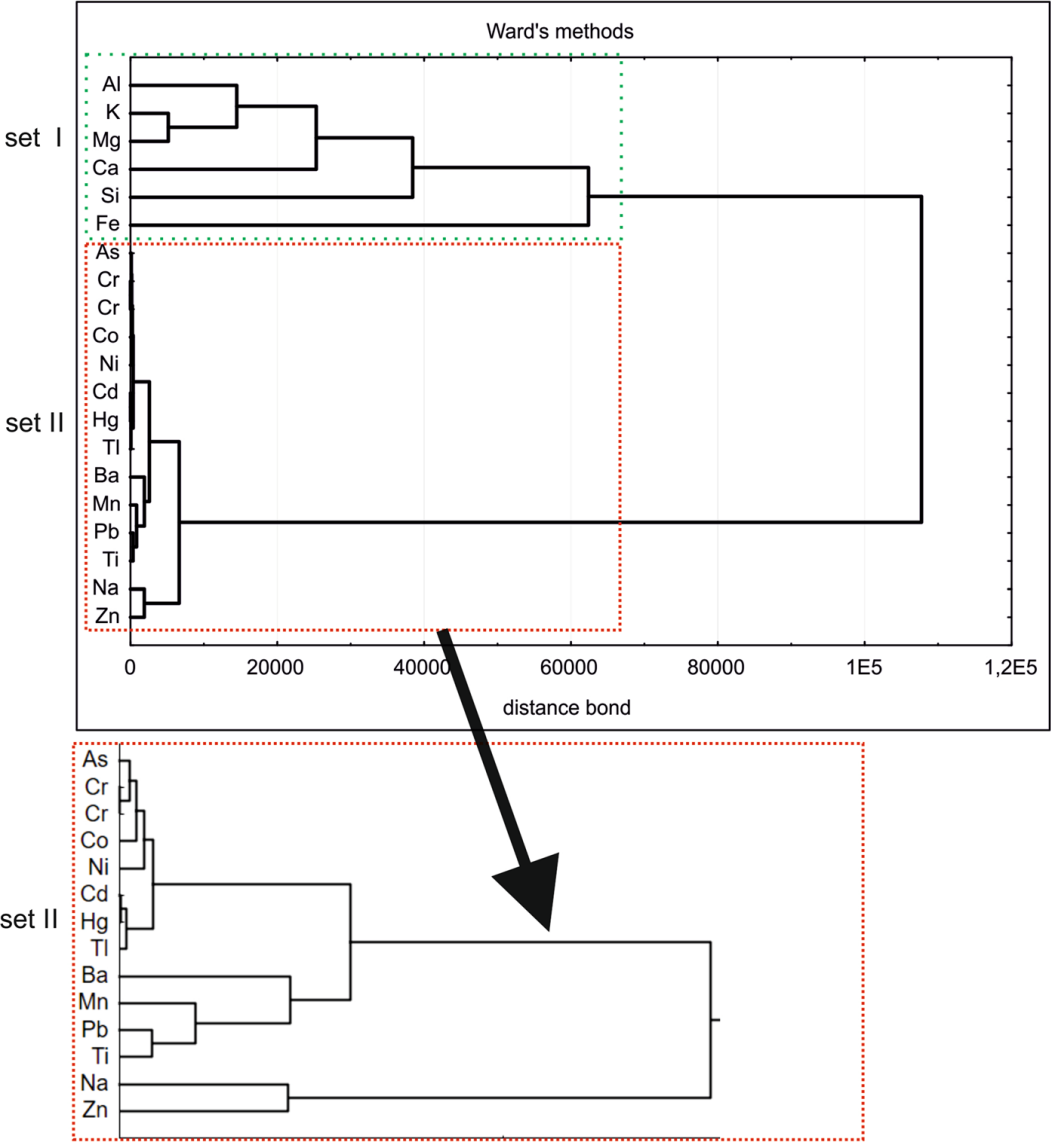
Dendrogram for specified elements content in urban atmospheric dust samples from Cracow

The results of chemical determinations were subjected to cluster analysis in order to separate elements that form uniform statistical sets of their population in airborne dusts (Fig. 7). Two distinct sets have been distinguished.

The set I is composed of Al, K, Mg, Ca, Si and Fe, which can be considered geogenic elements. Their presence results from wind-blowing (quartz) and weathering (mainly aluminosilicates) of various primary minerals, and in the case of Fe its anthropogenic contribution should be recognized because of the proximity of the steel works to the park in Nowa Huta. The set II consists of As, Cr (the two strongly related to each other), Co and Ni. Their source is anthropogenic, most often attributed (Mazzei et al. 2008) to the movement of vehicles. The presence of Cd, Hg and Tl is a result of both the car fuel combustion and the low emission. The remaining elements: Ba, Mn. Pb, Ti, Na and Zn may have their sources in the operations of the Arcelor steel plant of Krakow and also of other metallurgical facilities located some 35 km to the west (processing Zn–Pb ores in Bukowno–Olkusz) and even of such facilities located farther to the west in Upper Silesia (80 km). The western direction is important to be mentioned as it is the prevalent direction of winds recorded in Krakow.

## Health risk assessment

High concentrations of the contaminants in the dusts (Table 2) indicate two kinds of hazards endangering health and even life of Krakow residents. The toxins may enter the human organism mainly by inhaling the finest dusts of the <PM_2.5_ group and from lungs are distributed with blood into other organs. Additional amounts of deleterious elements can be absorbed by the children playing and walking in the open, mainly by putting dirty hands into their mouths.

To obtain comparative numerical data, certain assumptions based on the reports of the World Health Organization (WHO) (1982, 1993, 1994) and US Environmental Protection Agency (EPA) (1986) recommendations were accepted. They served to establish average daily doses (ADD) of toxic elements contained in the urban dusts, from which the hazard quotient (HQ) values were calculated (Table 3) according to the expression (1). The health risk has been assessed for two age groups: children (6 years old, with the body mass 15 kg) and adults (70 years old, with the body mass 70 kg). The HQ values of the two groups considerably differ, due mainly to the lower body mass of children. The health risk of the youngsters exposed to high trace metal concentrations in the park dusts has been ranked in the following order of the HQ values: As = 3.19E+00 > Tl = 2.79E+00 > Fe = 8.86E−01 ≅ Pb = 6.96E−01. The HQ values of the remaining elements considered indicate low health risk.

**Table 3:** Hazard quotient (HQ*)* and average daily dose *(ADD)* calculated for adults and children

Element	RfD^a^	Children BW = 15 kg, ED = 6		Adults BW = 70 kg, ED = 70	
		ADD (mg/kg/day)	HQ	ADD (mg/kg/day)	HQ
Al	1.00E+00	6.78E−02	6.78E−02	7.26E−03	7.26E−03
As	3.00E−04	9.59E−04	**3.19E+00**	1.03E−04	3.42E−01
Ba	7.00E−02	5.76E−03	8.22E−02	6.17E−04	8.81E−03
Cd	1.00E−03	7.67E−05	7.67E−02	8.49E−06	8.49E−03
Co	2.00E−02	5.47E−04	2.70E−02	5.86E−05	2.93E−03
Cr	3.00E−03	8.22E−04	2.74E−01	8.81E−05	2.94E−02
Fe	3.00E−01	2.78E−01	8.86E−01	2.98E−02	9.49E−02
Hg	3.00E−04	1.94E−05	6.50E−02	2.08E−06	6.92E−03
Mn	4.60E−02	5.97E−03	1.30E−01	6.39E−04	1.39E−02
Ni	2.00E−02	7.77E−04	3.90E−02	8.33E−05	4.16E−03
Pb	3.50E−03	2.43E−03	6.95E−01	2.61E−04	7.445E−02
Si	4.20E−01	9.91E−02	2.36E−01	1.06E−02	2.53E−02
Ti	1.00E+00	2.86E−03	2.86E−03	3.07E−04	3.07E−04
Tl	8.00E−05	2.24E−04	**2.79+00**	2.40E−05	3.00E−01
Zn	3.00E−01	1.22E−02	4.10E−02	1.31E−03	4.37E−03

According to WHO (1982, 1993, 1994)

*BW* body mass, *ED* exposure duration, bolded HQ exceeding one unit

^a^RfD (reference dose) according to IRSIS US EPA (1986) or calculated as per cent of PTWI (permitted tolerable weekly intake)

The fact that almost all of the elements considered form compounds that are highly toxic and also cause cancerous changes of organisms exposed to their presence is even more disturbing (IARC 1990). Arsenic (As^3+^ and As^5+^) belongs to the group I of carcinogenic elements according to the International Agency for Research on Cancer IARC (1987), and its long-lasting nutritional and inhaling exposition results in skin changes and affects functioning of the circulatory, nervous and respiratory systems (Kabata-Pendias and Szteke 2012). Thallium has a negative impact on the heart and also on the digestive, nervous and respiratory systems. It is a cellular poison (Kabata-Pendias and Szteke 2012) disturbing the cellular equilibrium and results in a general poisoning of the organism.

The HQ values applicable to adults are of a lower health impact those of children. The results indicate a possibility of adverse health effects of As (HQ = 3.42E−01) and Tl (HQ = 3.00E−01) carried by urban dusts. If a daily dose of 200 mg of the accidentally swallowed soil or dirt containing urban dusts is unquestionable for children, a dose of 100 mg for adults seems to be slightly overestimated.

It should be stressed that the calculations have omitted the metals entering the human body through the skin or via the inhaling system. Due to that, the real health risk must be considerably higher than the one presented here.

## Discussion

The health impact caused by the trace metals potentially toxic to people exposed to dust in urban parks of Krakow and expressed by the HQ index (Table 3) shows that the HQ values for children and adults decrease in the same order: As > Tl > Fe > Pb > Cr > Mn. The HQ_ing_ values of As and Tl for children are 3.19E+00 and 2.79E+00, respectively, whereas for adults they are considerably lower, around ten times: 3.42E−01 and 3.00E−01, respectively. It is an effect of a smaller, accidental intake of these elements accepted in the calculations (100 mg by adults, 200 mg by children) and a higher body mass of adults. The HQ_ing_ values of As and Tl calculated for children of Krakow are significantly higher than those of other large agglomerations (Table 4), e.g. 1.13E−01 As established in Luanda (Ferreira-Baptista and De Miguel 2005), 6.32E−02 As and 1.29E−02 Tl in Madrid (Miguel et al. 2007), and 8.71E−02 As in Nanjing (Wang et al. 2016).

**Table 4:** Comparison of average total content metals in dust in different cities and health risk from metals

Location	Al	As	Ba	Cd	Co	Cr	Hg	Mn	Ni	Pb	Tl	Zn	
Beijing, China^a^	TC	–	–	–	0.64	–	69.33	–	–	25.97	201.82	–	219.20
	HQ_ing_ Ch HQ_ing_ Ad	–	–	–	8.81E−03 1.10E−03	–	7.72E−01 2.38E−03	–	–	1.66E−02 2.23E−03	7.37E−01 9.89E−02	–	9.33E−03 1.25E−03
Nanjing, China^b^	TC	–	17.3	–	1.92	11.5	133	–	602	115	119	–	585
	HQ_ing_ Ch HQ_ing_ Ad	–	8.71E−02 1.14E−02	–	8.20E−03 1.08E−03	–	2.60E−02 3.41E−03	–	4.51E−02 5.19E−03	9.15E−03 1.20E−03	1.32E−01 1.73E−02	–	6.18E−03 8.10E−04
Luanda, Angola^c^	TC	4839	5	131	1.1	2.9	26	0.13	258	10	351	–	317
	HQ_ing_ Ch	3.33E−02	1.13E−01	1.30E−02	7.91E−03	1.00E−03	5.80E−02	3.15E−03	3.86E−02	3.52E−03	7.10E−01	–	7.30E−03
Madrid, Spain^d^	TC	7570	7.3	86	0.19	3.6	20	0.24	285	6.9	38	0.16	78
	HQ_ing_ Ch	1.94E−02	6.32E−02	3.37E−03	5.51E−04	–	1.81E−02	2.24E−03	1.66E−02	9.27E−04	2.92E−02	1.29E−02	6.77E−04
Krakow, Poland	TC^e^	5300	75	450	6	43	64	1.5	467	61	190	17	956
	HQ_ing_ Ch HQ_ing_ Ad	6.78E−02 7.26E−03	3.19E+00 3.42E−01	8.22E−02 8.81E−03	7.67E−02 8.49E−03	2.70E−02 2.93E−03	2.74E−01 2.94E−02	6.50E−02 6.92E−03	1.30E−01 1.39E−02	3.90E−02 4.16E−03	6.95E−01 7.44E−02	2.79+00 3.00E−01	4.10E−02 4.37E−03

TC—total content metals (mg/kg), HQ_ing_ Ch*—*health risk ingestion for children, HQ_ing_ Ad*—*health risk ingestion for adults

^a^Du et al. (2013)

^b^Wang et al. (2016)

^c^Ferreira-Baptista and De Miguel (2005)

^d^Miguel et al. (2007), TC*—*data for year 2002

^e^This study

The HQ_ing_ values of Al, Ba, Cd, Co, Fe, Hg and Mn obtained for the inhabitants of Krakow are higher by at least one order of magnitude from the figures published by Ferreira-Baptista and De Miguel (2005) for Luanda, Wang et al. (2016) for Nanjing, Du et al. (2013) for Beijing, and Miguel et al. (2007) for Madrid. In turn, higher than in Krakow health risk values for children were found in Beijing 7.72E−01 of Cr (Du et al. 2013) and in Nanjing 7.37E−01 of Pb (Wang et al. 2016), whereas the higher HQ_ing_ value than for Krakow adults Beijing (Du et al. 2013) only in the case of Pb 9.89E−02.

High concentrations of As, Tl, Zn and Cd in the dusts of Krakow are associated without doubts with long-distance emissions from the Olkusz area (around 40 km west of Krakow), where lead and zinc deposits were mined as early as the twelfth century. The Olkusz emissions contain high quantities of As, Cd, Tl and other, often toxic elements and contaminate the air and the soil–plant system not only in the closest neighbourhood (Kicinska and Gruszecka-Kosiwska 2016).

The quantities of Cr in the dusts of Krakow are more than 2 times lower than those given by Wang et al. (2016) in Nanjing and comparable to those in Beijing (Du et al. 2013). They exceed 2–3 times the values determined in Luanda (Ferreira-Baptista and De Miguel 2005) and Madrid (Miguel et al. 2007). The quantities of Mn and Ni in the dusts of Nanjing (Wang et al. 2016) exceed those of Krakow. The quantities of Pb in the dusts of Luanda (Ferreira-Baptista and De Miguel 2005) are considerably higher than those of Krakow. The dust contents of Al in Madrid (Miguel et al. 2007) exceed only 1.5 times those noted in Krakow.

Another issue to be solved is the quantity of metals in the remaining seasons. The recalculation of the mean annual concentrations of elements in the air (Samek et al. 2016, see Table 2) indicates that they approximate most the spring mean concentrations (the only exception is Ca that dominates in the spring months). Therefore, it can be assumed that sampling carried out in the spring will give the most reliable and representative results for a town located in the same part of Europe as Krakow and with a moderate climate. As a result, the HQ index will be considerably higher during winter season than that determined in the summer. It must be remembered, however, that the winter time in this part of Europe does not favour playing in urban parks, thus assuming in calculations 2-h stays of children in playgrounds are definitely too high. The results obtained by Goudarzi et al. (2016) in the areas of the dry climate for sulphur compounds and airborne dusts are different than ours and indicate that the autumn is a better comparable period than spring.

## Conclusions

The research material included urban dusts collected in three parks of Krakow, an agglomeration of a million of inhabitants. Their mineralogical and chemical analyses have provided the following conclusions.

1. The airborne dusts are considerably diversified considering their morphology, structural development, grain-size distribution and chemical composition.
2. Prevalent mineral phases include quartz and feldspars (geogenic minerals), also unburned coal remains (low emissions from households and small boiler houses) and gypsum (secondary phase of the anthropogenic origin). Other anthropogenic particles are metallic spherules, generated in metallurgical processes.
3. The presence of various hydrocarbons in the dusts of the three parks considered: i.e. of mineral oils and traces of benzines, results from combustion of motor fuels.
4. The chemical composition of the dusts, particularly their contents of Cd, Ba, Ti, Pb, Zn and As, indicates a prevalently anthropogenic origin of contaminants.
5. High concentrations of heavy metals of the dusts are a significant factor of air pollution. The dust fallouts spread pollutants to soils, plants and surface waters and also directly affect the health of Krakow inhabitants.
6. The health quotients HQ > 10 are a clear sign of a chronic exposure of children playing and walking in the Krakow Parks to As, Cr, Co, Ti and Tl. All these elements are highly toxic to humans, particularly to young organisms with a low body mass.
